# Sleep and Delirium: Understanding Links, Mechanisms, and Associations with Neurodegenerative Conditions

**DOI:** 10.1002/alz.090822

**Published:** 2025-01-03

**Authors:** Andrew Krystal

**Affiliations:** ^1^ University of California San Francisco, San Francisco, CA USA

## Abstract

Those with neurodegenerative conditions have an increased risk of developing delirium and there is some evidence that delirium may be a risk factor for neurodegenerative disorders. Similar to the interactions between sleep and neurodegenerative conditions, there is increasing evidence for bi‐directional relationships between delirium and sleep disorders and disturbances. This presentation will provide an overview of the literature on those relationships, including discussion of the well‐characterized changes in sleep that occur in individuals experiencing delirium. Evidence that sleep disorders and disturbed sleep increase the risks of delirium and that treatments addressing sleep disorders decrease those risks will also be reviewed. Studies have demonstrated that both obstructive sleep apnea and sleep disturbance significantly increase the risk of post‐operative delirium, and post‐operative delirium is associated with an increased risk of cognitive decline and possibly dementia There is a growing set of studies assessing the degree to which pharmacologic therapies that address disturbed sleep, including dual orexin receptor antagonists, melatonin, and melatonin receptor agonists, mitigate those risks. Studies of the effects of behavioral interventions aimed at diminishing sleep disturbance in the hospital will also be discussed. In addition, studies assessing whether treating obstructive sleep apnea with continuous positive airway pressure therapy decreases risks for the emergence of delirium will be reviewed. Implications of these studies for understanding the mechanisms by which sleep and delirium are linked and which might explain why neurodegenerative conditions increase the risks for delirium will also be considered. Lastly, the need for future efforts will be presented including rigorous mechanistic studies, larger well‐controlled trials of treatments and development of clinical care paths.

